# Genetic variation in zona pellucida-3 (ZP3) gene and its association with litter size variation in Kari sheep

**DOI:** 10.1080/10495398.2025.2450364

**Published:** 2025-01-24

**Authors:** Izaz Ali, Muhammad Ibrahim, Sohail Ahmad, Sher Hayat Khan, Ihtesham Ul Haq, Ibrahim A. Alhidary, Rifat Ullah Khan, Naseer Khan Momand, Marco Ragni

**Affiliations:** a Animal Biotechnology Division, Institute of Biotechnology and Genetic Engineering, The University of Agriculture, Peshawar, Pakistan; b Department of Animal Production, College of Food and Agriculture Science, King Saud University, Riadh, Saudi Arabia; c College of Veterinary Sciences, Faculty of Animal Husbandry & Veterinary Sciences, The University of Agriculture, Peshawar, Pakistan; d Nangarhar University, Jalalabad, Afghanistan; e Department of Plant, Soil and Food science, University of Bari, Aldomoro, Italy

**Keywords:** Genetic variation, zona pellucida-3 (ZP3), Kari sheep, litter size, reproductive efficiency

## Abstract

Variation in litter size (LS) in sheep is linked to genetic factors, including the Zona pellucida-3 (ZP3) gene, which plays a role in ovine reproductive processes. This study examined the association between ZP3 gene variations and LS in Kari sheep. Two groups of 160 Kari ewes were analysed: one consistently producing singletons and another producing twins, with occasional triplets. Additionally, Madakhlasht sheep, which sometimes produce twins, and Balkhi sheep, which produce only singletons, were used as references. The entire ZP3 gene was amplified using PCR and sequenced at 30× with Next Generation Sequencing. Bioinformatics analysis identified 70 variants across the three breeds, located in upstream regions, introns, and exons. Notably, two point mutations and a six-nucleotide insertion were found upstream of the initiation codon in twin-producing Kari ewes, potentially affecting ZP3 expression and LS. Two missense mutations (I101L in exon 1 and R408H in exon 8) were heterozygous in twin-producing Kari ewes but homozygous in other groups, correlating with LS. Protein modelling suggested that the I101L mutation alters the binding site, potentially impacting protein function. These findings indicate that ZP3 gene variations influence reproductive efficiency and LS in sheep, with specific variants serving as potential markers for selective breeding to enhance LS.

## HIGHLIGHTS

- ZP3 gene variants link to litter size in Kari sheep, impacting reproductive efficiency.
- Twin-producing Kari ewes show unique upstream mutations affecting ZP3 expression.
- Missense mutations in ZP3 correlate with litter size, suggesting potential for selective breeding.

## Introduction

Surrounding mammalian eggs is a porous, transparent glycoprotein coat known as the zona pellucida (ZP), comprising four glycoproteins designated as ZP1 to ZP4.^1^^,^^2^ This glycoprotein membrane plays a pivotal role in the initial development and maturation of ova as well as in fertilization.^3^ It possesses species-specific receptors for spermatozoa, which facilitate the acrosome reaction.^4^^,^^5^ During fertilization, molecular changes in the zona pellucida membrane prevent the entry of more than one sperm.^3^^,^^6^^,^^7^ Among the four ZP glycoproteins, ZP3 contains the initial receptors for sperm, facilitating the early binding of sperm with the egg and initiating the acrosomal reaction.^8–10^ After fertilization, ZP3 loses its sperm receptor activity and becomes incapable of inducing the acrosomal reaction.^11^^,^^12^

Sheep are a vital component of agricultural systems worldwide, valued for their wool, meat, and milk, and their adaptability to diverse environments.^13–16^ Productivity is a significant financial and economic aspect of sheep farming,^17^^,^^18^ with litter size being a crucial reproductive trait targeted for improving reproductive efficiency. Studies have linked sequence polymorphisms in the zona pellucida gene 3 to litter size variation in sheep.^19^ For instance, in Hu sheep, litter size at first parity averages 1.72 lambs per lambing, averaging 2.17 lambs per birth, the litter size for second parity ewes shows a notable increase.^20^ Comparable phenotypes have been documented in various other sheep breeds, such as Swiss sheep, with litter sizes ranging from 1.36 to 1.57 in the first parity and 1.52 to 1.75 in subsequent parities.^21^ These findings suggest that litter size in the first parity acts as a limiting reproductive trait in sheep. Research examining sequence variations in the ZP3 gene has identified a single nucleotide polymorphism (SNP) at position rs401271989 that could affect the litter size at first parity in Chinese Hu sheep, potentially serving as a useful marker for selecting desired litter sizes.^22^

The majority of current studies investigating the zona pellucida membrane and its impact on reproductive performance focus on species other than sheep. Mutations in genes encoding ZP glycoproteins have been demonstrated to affect reproduction in humans and other animals.^23^ These mutations often result in infertility in humans, leading to various conditions such as ZP-free oocytes,^24^^,^^25^ diminutive ova,^26^ and empty follicle syndrome.^27^

A study was conducted to document some traits that characterize the unique sheep breed locally named ‘Kari’ found in Garam Chashma valley of the Chitral district of Khyber Pakhtunkhwa, Pakistan. Kari is a lightweight breed with a thin tail, having no definite coat colour, but the white colour is principally (75%) found in the flocks. They have short ears, and males are usually horned while the females are hornless. Kari has an average adult body weight of 21.85 ± 0.11 kg for males and 18.34 ± 0.08 kg for females. The birth weight of lambs born single is 2.16 ± 0.04 kg which is 23.6% heavier than the ones born in twin (1.75 ± 0.05 kg). Kari sheep have a mean litter size of 1.2 ± 0.01 producing 3.88 ± 1.10 lambs per annum.^28^

Studying litter size in sheep and identifying genetic variants associated with its variation are crucial for the conservation and enhancement of sheep genetic resources. Previous studies have reported variation in litter size and genetic diversity in the Kari sheep breed from Chitral, Khyber Pakhtunkhwa.^28^^,^^29^ Therefore, the current study was designed to assess sequence variation in the ZP3 gene among sheep exhibiting different litter sizes and to analyse genetic variants linked to higher litter size in Kari sheep.

## Materials and methods

All the following experiments were performed according the guidelines laid by Pakistan Animal Welfare Society and with prior approval from the Ethical Committee of The University of Agriculture Peshawar, Pakistan (112/2022/IBGE).

### Animals

The data of reproductive performance were recorded for a period of two years (2018–2019) from 160 Kari sheep (aged 2–5 years) reared in a transhumant production system with continuous breeding. Among the 160 ewes, eighty were identified producing twins or triplets in each lambing. Another eighty Kari ewes producing singletons were selected as control group. These 16 sheep were analysed for sequence variation of ZP3 gene. Another two sheep breeds were also sampled to compare their results with Kari specimen, that is, Balkhi sheep always producing singleton and Madakhlasht sheep seldom producing twins.

### Sample collection and DNA isolation

Blood samples of 3 mL were collected using disposable syringes from the jugular vein of the sheep following standard biosafety protocol. The samples were transferred to prelabelled EDTA vacationer to prevent coagulation and kept immediately in an icebox. The samples were shifted to the laboratory where they were stored at −80 °C until further processing. Genomic DNA was extracted from blood samples using Nucleo Spin^®^ Blood genomic DNA purification kit (MACHEREY-NAGEL, Catalog No. 740951.250), following the manufacturer protocol. The quality of the genomic DNA was assessed by running it on a 1% agarose gel to ensure purity and integrity. Nanodrop reading was checked to assess the concentration of the extracted DNA. Only high-quality and intact DNA was used for PCR.

### Primer designing

Primers were designed using Primer 3 tool using fasta files of the sheep’s ZP3 gene taken from NCBI (Table 1). Primer blast, UCSC browser PCR tool, and BLAT tool were used to confirm primer’s specificity. Two sets of primers were designed to cover the whole 7.7 kb of the ZP3 gene along with a 1 kb upstream region.

**Table 1.** Primers designed to amplify ZP3 gene in indigenous sheep breeds.

Primer ID	Sequences (5’ to 3’)	GC%	Product size	TM	Annealing temp
*ZP3*-1F	CTCCTCATCCTTAATCAATCC	42.9	4597 bp	58.6	57.9
*ZP3*-1R	CAGGCTTATTCCAGGCTATTA	42.9		60.3	
*ZP3*-2F	ACCTCCTCCGTCTCTAACTG	55.0	4467 bp	62.5	61.2
*ZP3*-2R	CCTCTCTCCTCTTCGTTGTT	50.0		61.2	

### Polymerase chain reaction

High fidelity PCR master mix ABM Kodaq 2× (Cat no: G497 made in Canada) was used for amplifying the ZP3 gene using predesigned primers. A 15 µL reaction mixture was prepared, containing 1× master mix, 0.4 µM each of forward and reverse primers, and 100 ng of genomic DNA. The cycling conditions included an initial denaturation at 94 °C for 3 minutes, followed by 30 cycles of denaturation at 94 °C for 30 seconds, annealing at 58 or 61 °C for 30 seconds, and extension at 72 °C for 5 minutes. The reaction was followed by a final denaturation at 72 °C for 5 minutes. The PCR products were sequenced through next-generation sequencing using Illumina MiSeq system at Rehman Medical Institute Peshawar.

### Bioinformatics analysis

By using the FastQC tool (v0.11.8) the read qualities of fastq files were verified. Trimmomatic tool (v0.39) was used to eliminate soft-quality base calls (Q < 30) and index adapter sequences. Using Burrows-Wheeler Aligner (BWA, v0.6) algorithm the purified reads were allied to the sheep reference genome (Oar_rambouillet_v1.0, Accession # GCF_002742125.1). Using Picard tools (v2.21.6) the PCR duplicates were removed from the reads.

GATK’s ‘BaseRecalibrator’ was used for base quality score recalibration (BQSR) to repair base quality score estimates produced by various technical and organized sequencing items using default settings. Finally, GATK ‘Variant Filtration’ was applied to eliminate potential fake variants from the primitive variants callset.

### Protein modelling

For protein remodelling, the protein FASTA sequence was extracted, and amino acid changes were identified using the HOPE tool. Then, past these sequences in LOMET which form a 3D model of the ZP3 wild protein in PDB format. Then the missense 3D was used which identified the effect of amino acid changes.

### Statistical analysis

Means and standard errors were calculated for morphometric parameters among different sheep breeds. To address breed and parity differences, we used a mixed-effects model that includes breed, parity, and their interaction as fixed factors, with individual animal variability as a random factor using SPSS (version 23.0). Allelic polymorphism, heterozygosities, genetic distance, population differentiation, and markers neutrality were calculated for the observed SNPs using POPGENE software. Association between litter size and allelic polymorphism was analysed by performing principal component analysis (PCA) in SPSS software. Pearson’s correlations were calculated to analyse correlation between litter size and SNPs.

## Results

### Body size variation among breeds

**Figure 1.**
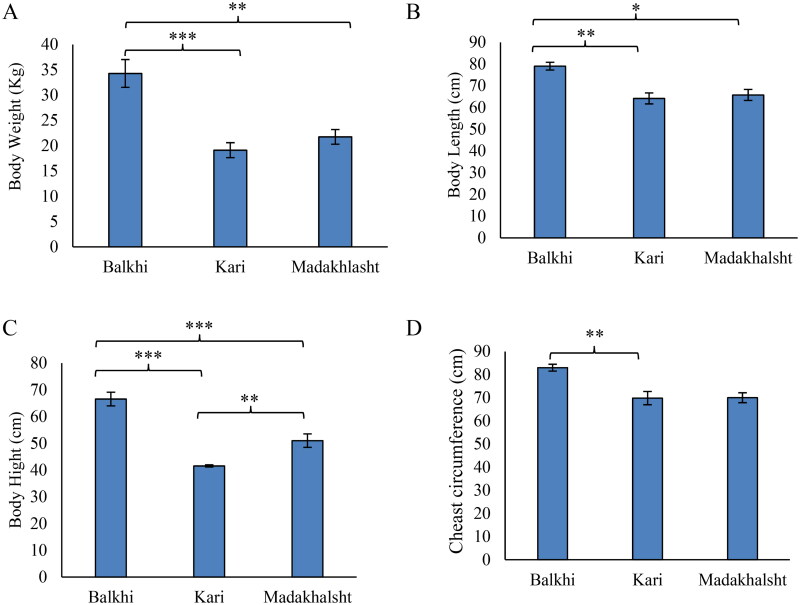
Body size parameters of the three sheep breeds measured using a weighing balance and measuring tape. (A) Bodyweight. (B) Body length. (C) Body height. (D) Chest circumference. Bars represent the mean value. Error bars represent standard error. The number of observations was 16 for Balkhi, 16 for Kari, and 14 for Madakhlasht. *** = p < 0.001; ** = p < 0.01; * = p < 0.05 (Post Hoc Tukey’s test).

The morphological measurements of the body size of the three sheep breeds having variation in litter size are presented. Among the three sheep breeds, Balkhi was the significantly heavier than Kari and Madakhlasht sheep breeds having a bodyweight of 34.3 ± 2.8 kg (Figure 1(A)). Body length of Balkhi sheep was 79 ± 1.8 cm, which was significantly longer than Kari and Madakhalsht sheep breeds (*p* < 0.01 and *p* < 0.05, respectively) (Figure 1(B)). Balkhi was also significantly taller (*p* < 0.001) among the three breeds, having a body height of 66.5 ± 2.6 cm (Figure 1(C)). Chest circumference was also larger in Balkhi sheep (83 ± 1.5 cm) compared to Kari and Madakhlasht sheep breeds (Figure 1(D)).

**Figure 2.**
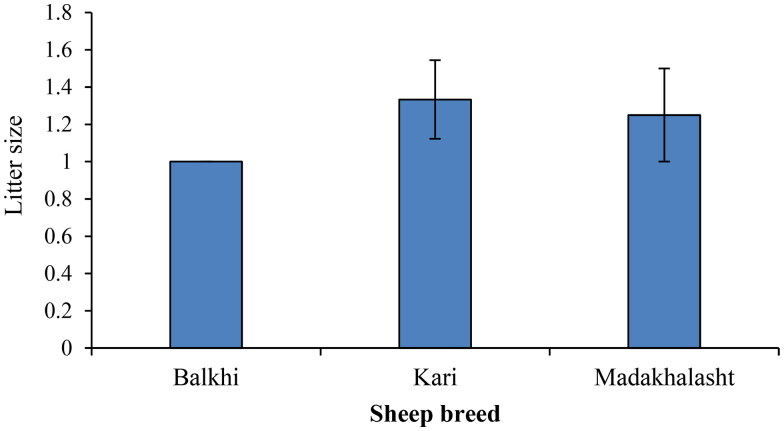
Mean litter size of the three sheep breeds calculated from the lambing record of the sheep resource center. Bars represent the mean value. Error bars represent standard error. The number of observations was 16 for Kari sheep, and 10 for each Balkhi and Madakhlasht sheep breeds.

### Variation in litter size among the sheep breeds

Mean litter sizes of the three sheep breeds selected for the current study is presented in Figure 2. Among the three sheep breeds, Kari sheep showed a higher litter size (1.33 ± 0.21), followed by the Madakhlasht sheep (1.25 ± 0.25). Balkhi sheep breed showed a litter size of one, which means that this breed produced exclusively one lamb per lambing. No significant difference was observed in the litter size of the three sheep breeds (Figure 2).

### Udder morphology

**Figure 3.**
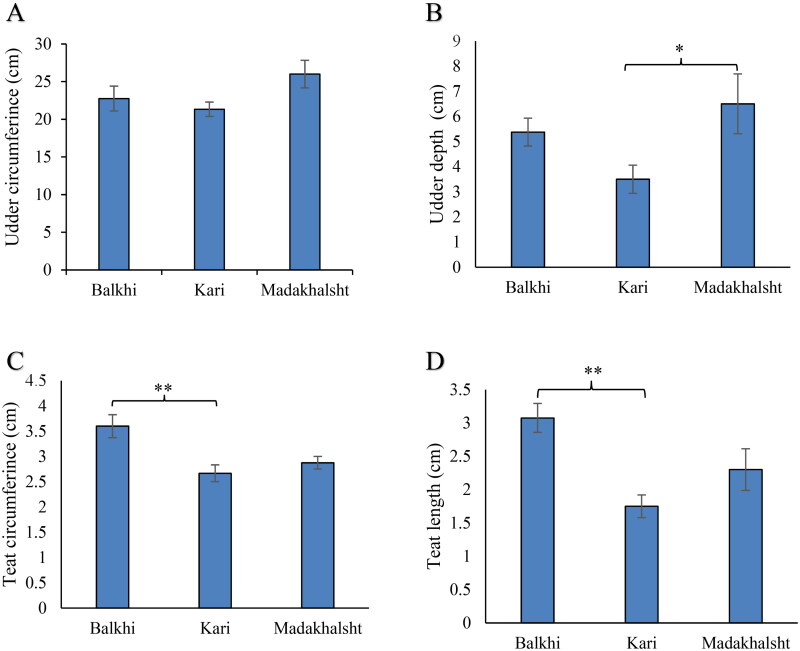
Udder morphometry of three sheep breeds measured using a measuring tape. Bars represent mean values in cm. Error bars represent standard error. The number of observations was 16 for Balkhi, 16 for Kari, and 14 for Madakhlasht. ** = p < 0.01; * = p < 0.05 (Post Hoc Tukey’s test).

The morphological measurements of udder size of the three sheep breeds having variation in litter size are presented in Figure 3. Among the three breeds, the udder circumference of Madakhalsht (26.0 ± 1.8 cm) was greater than Balkhi and Kari; however, the differences were not significant (Figure 3(A)). Madakhalsht sheep also showed greater value for udder depth (6.5 ± 1.2 cm) than Balkhi and Kari (Figure 3(B)). The udder depth in Madakhalsht sheep was significantly greater compared to Kari sheep (*p* < 0.05). The difference in the udder depth between Madakhalsht and Balkhi was not significant. Balkhi and Kari sheep breeds also showed statistically similar values for the udder depth. The teat circumference in Balkhi sheep was higher (3.6 ± 0.20 cm) among the three breeds (Figure 3(C)). Kari and Madakhalsht sheep breed showed similar teat circumference. Balkhi sheep have teat length (3.08 ± 0.2 cm) significantly (P < 0.01) longer than Kari and Madakhalsht (Figure 3(D)).

### Effect of body size of sheep on litter size

The differences in body size of the sheep breeds having variation in litter size are presented in Table 2. The results showed that bodyweight, body length, and body height of the sheep having litter size 1 were less than the measurements of the sheep having litter size 2; however, the differences were not significant. Similarly no significant differences were observed in the udder and teats measurements among sheep producing different litter size (Table 2).

**Table 2.** Variation in body morphology of Kari and Madakhlasht sheep breeds having different litter size.

Parameter	litter size		*P*-value ANOVA
	1	2	
Number of observations	8	8	
Body Weight	19.9 ± 1.4	20.7 ± 2.1	0.77
Body length	64.2 ± 2.2	66.0 ± 3.0	0.67
Height	46.5 ± 2.5	42.6 ± 0.6	0.37
Chest circumference	70.6 ± 2.4	68.3 ± 3.2	0.61
Abdominal circumference	82.7 ± 1.8	83.3 ± 5.3	0.9
Udder circumference	24 ± 1.6	21.3 ± 0.6	0.32
Udder depth	4.9 ± 0.9	4.3 ± 1.3	0.76
Teat length	1.9 ± 0.2	2.0 ± 0.3	0.91
Teat circumference	2.6 ± 0.2	3 ± 0.22	0.16

### PCR results

**Figure 4.**
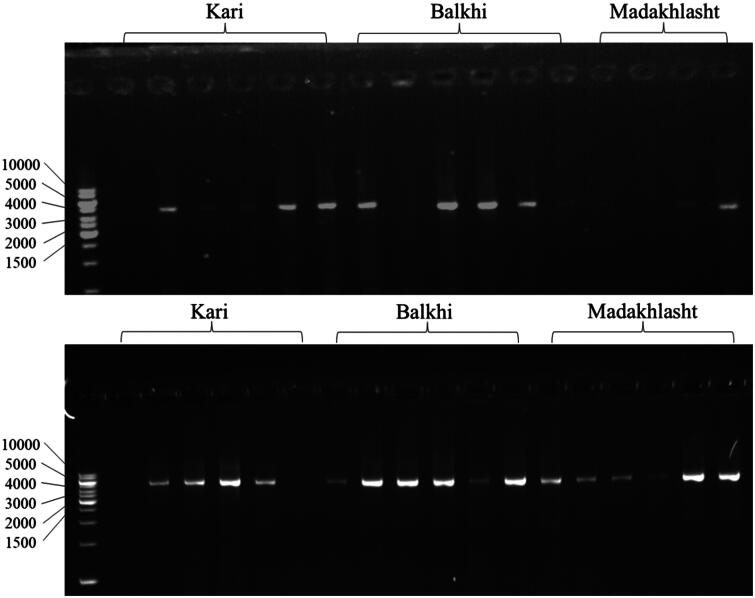
Gel picture of the PCR results of the ZP3 gene amplified using primer 1 (above, 4597 bp) and primer 2 (below, 4467 bp) conducted in 1.2% agarose gel. The ladder was 1 kb in the first well, the PCR products for Kari, Balkhi and Madakhlasht sheep breeds are labeled at the top of each gel.

The gel picture of the PCR products of ZP3 gene of 18 samples of Kari, Balkhi, and Madakhlasht sheep breeds is shown in Figure 4 using different primer sets covering the whole gene. The primer1 amplified a 4597 bp region of the ZP3 gene including the upstream region, exons 1–5, and introns 1–4. While primer 2 amplified the remaining 4467 bp of the ZP3 gene including introns 5–7, exons 6–8, and the downstream region. For each breed, six samples were taken. These PCR products were further sequenced using NGS.

### Gene variants in ZP3 gene

The NGS analysis of the ZP3 gene revealed a total of 70 variants across the three sheep breeds (Table 3). The majority (74%) of these variants were located in the intronic regions, with Balkhi having the most (44 variants), followed by Madakhlasht (42 variants), and Kari with 36 and 35 variants for litter sizes 1 and 2, respectively. The upstream region was the second most polymorphic area, accounting for 13% of the total variants. Kari with litter size 2 had the most upstream variants (7), followed by Madakhlasht (6), Balkhi (5), and Kari with litter size 1 having the least (3). A unique intronic variant affecting the splice region was observed only in Balkhi sheep. The coding region (exons) of the ZP3 gene also showed a total number of eight variants, of which synonymous variants were five, while a total of three missense variants changing the amino acid sequence of the ZP3 protein were found.

**Table 3.** Total variants observed in the ZP3 gene of the three sheep breeds compared to the reference genome.

Region	Kari		Madakhlasht	Balkhi	Total
	Litter size 1	Litter size 2			
Upstream	3	7	6	5	9
Intron	36	35	42	44	52
Synonymous	3	4	3	3	5
Splice region	0	0	0	1	1
Missense	3	3	3	2	3
Total	45	49	54	55	70

### Unique genotypes

Genotypes were assigned to each variant based on the heterozygosity or homozygosity of the variant in different sheep breeds. A total of 44 unique genotypes were observed in the three sheep breeds (Table 4). The highest number of unique genotypes were observed in Kari sheep having litter size 1, most of which were the homozygous forms of mutations that were found in heterozygous forms in the other category sheep. The highest number of unique (homozygous) genotypes (24) in Kari having litter size 1 were observed in the intronic region. Kari with litter size 2 showed a total number of six unique genotypes of which three were in the upstream and three in the intronic region. Madakhlasht sheep have four and Balkhi have three unique genotypes in the intronic region. Balkhi sheep also showed one unique genotype for the synonymous mutation and one for the mutation at the splice region site.

**Table 4.** Unique genotypes observed in the ZP3 gene among the three sheep breeds.

Region	Kari		Madakhlasht	Balkhi	Total
	Litter size 1	Litter size 2			
Upstream	1	3	0	0	4
Intron	24	3	4	3	34
Synonymous	3	0	0	1	4
Missense	1	0	0	0	1
Splice region	0	0	0	1	1
Total	29	6	4	4	44

### Shared genotypes

Shared genotypes among the four sheep categories (Kari having litter size 1, Kari having litter size 2, Madakhlasht, and Balkhi) are presented in Table 5. The highest number of genotypes (44) were shared between Balkhi and Madakhlasht. Kari having litter size 1 shared the least number of genotypes with other sheep categories including Kari having litter size 2. Interestingly, Kari having litter size 2 shared more genotypes with Madakhlasht and Balkhi than it shared with Kari having litter size 1. The detailed results of genotype sharing between breeds at different genetic regions are presented in Table 5.

**Table 5.** Shared genotypes at different regions of the ZP3 gene among the three sheep breeds.

Region		Kari1	Kari2	Madakhlasht
Upstream	Kari2	2		
	Madakhlasht	0	2	
	Balkhi	0	0	5
Intron	Kari2	7		
	Madakhlasht	6	22	
	Balkhi	4	28	35
Synonymous	Kari2	0		
	Madakhlasht	0	3	
	Balkhi	0	3	2
Missense	Kari2	1		
	Madakhlasht	1	3	
	Balkhi	1	2	2
Total	Kari2	10		
	Madakhlasht	7	30	
	Balkhi	5	33	44

### Upstream gene variants

A total of nine upstream variants were identified in the ZP3 gene across indigenous sheep breeds (Table 6). The number of upstream variants varied among the breeds. Kari with litter size 2 exhibited the highest number of upstream variants, with a total of 7. The least number (3) of upstream variants were observed in Kari with litter size 1. An insertion of seven nucleotides in the TATA box of the upstream region was observed only in Kari having litter size 2, the remaining 8 variants in the upstream region were SNPs. Beside the insertion in Kari having litter size 2 at genomic position 35135200, two other SNPs in the upstream region were unique to this category. Madakhlasht and Balkhi sheep shared the greatest number of variants in the upstream region of the ZP3 gene. Mutation frequency was higher at two loci 35134586 and 35134939, these mutations were observed in all the indigenous sheep breeds. Two SNPs in Kari having litter size 2 and three SNPs in Kari having litter size 1 were homozygous. All the remaining variants in the upstream region of the three sheep breeds were in heterozygous forms.

**Table 6.** Upstream modifier variants in the three sheep breeds compared to the reference genome. Proximal promoter response elements (control expression in response of stimuli).

Genomic Position	Reference	Kari		Madakhlasht	Balkhi	Mutation frequency
		Litter size 1	Litter size 2			
35134572	T		C	C		0.25
35134586	G	A	A	A	A	0.75
35134916	T			C	C	0.25
35134939	A	G	G	G	G	0.75
35134943	G			C	C	0.25
35135041	A	G	G	G	G	0.625
35135200	T		TGATTAA			0.125
35135203	C		A			0.125
35135236	G		A			0.125

Grey boxes indicate homozygous mutations.

### Missense variants

A total number of three missense variants were observed in the ZP3 gene of indigenous sheep breeds (Table 7). Different sheep breeds showed variations in the number of missense variants. Kari sheep breed with litter sizes 1 and 2 and Madakhlasht sheep breed showed the highest number of missense variants (three). The least number (two) of missense variants were observed in Balkhi. Madakhlasht and Kari sheep with litter size 2 shared the greatest number of missense variants in the ZP3 gene. Mutation frequency was higher at one locus 35135618, which showed a mutation in all the indigenous sheep breeds. Amino acid change (Leu9Phe) and (Ile101Leu) was observed at loci 35135342 and 35135618 in all the breeds, similarly, a change in the amino acid sequence (Arg408His) was only observed in breed Kari and Madakhlasht at locus 35142968.

**Table 7.** Missense variants in the coding region of the ZP3 gene changing the amino acid sequence.

Genomic position	Reference	Kari		Madakhlasht	Balkhi	Mutation frequency	Amino acid change
		Litter size 1	Litter size 2				
35135342	C	T	T	T	T	0.50	p.Leu9Phe
35135618	A	C	C	C	C	0.625	p.Ile101Leu
35142968	G	A	A	A		0.50	p.Arg408His

Grey boxes indicate homozygous mutations.

### Intron variants

Fifty-two different intron modifier variants were identified in the ZP3 gene of native sheep breeds (Supplementary Table 1). A different number of intron modifier variants was observed in different sheep breeds. Balkhi showed the highest number of intron modifier variants (44). The least number (35) of intron modifier variants were observed in Kari sheep having litter size 2. Twenty-four unique genotypes at genomic positions 35135652-35142675 were observed in the intronic region of Kari having litter size 1. Madakhlasht and Balkhi sheep shared the most number (35) of variants in the intronic region of the ZP3 gene. Mutation frequency was higher at locus 35136952, which showed a mutation in all the indigenous sheep breeds.

### Synonymous and splice variants

A total of six low-impact variants were found in the ZP3 gene of the three sheep breeds. Five of these variants were synonymous, affecting the coding region in such a way that the amino acid sequence of the resulting protein does not change. One low-impact variant was found in the splice region of intron 7 (Table 8). A different number of synonymous variants was observed in different sheep breeds. Kari with litter size 2 and showed the highest number (4) of synonymous. Balkhi, Madakhlasht, and Kari having litter size 1 showed three synonymous variants each; however, the positions of these variants were different. One splice region variant was found only in Balkhi sheep. Mutation frequency was higher for the synonymous mutation at locus 35135437 (0.625), which showed a mutation in all the indigenous sheep breeds. Two synonymous mutations were homozygous in Kari having litter size 1, the rest of the mutations were in heterozygous form.

**Table 8.** Synonymous and splice region variants among different sheep breeds.

Category	Genomic position	Reference	Kari		Madakhlasht	Balkhi	Mutation frequency
			Litter size 1	Litter size 2			
Synonymous	35135437	C	T	T	T	T	0.625
	35135554	G		A		A	0.250
	35138759	C	T				0.125
	35141765	G	A	A	A		0.50
	35142934	C		T	T	T	0.375
Splice region	35141927	T				C	0.125

Grey boxes indicate homozygous mutations.

### Genetic distance among the three sheep breeds based on ZP3 gene polymorphism

**Figure 5.**
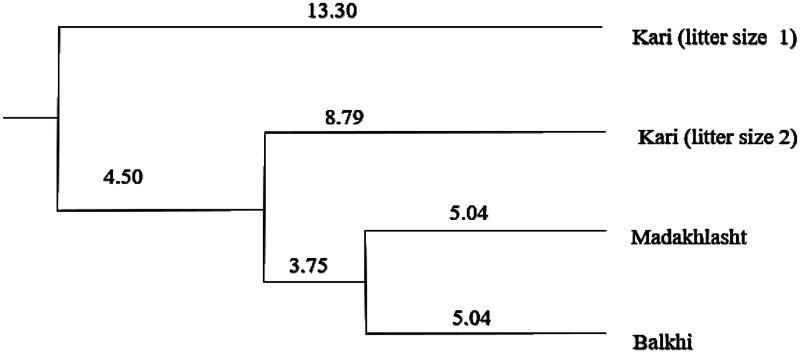
Dendrogram showing genetic distance among three sheep breeds and between the Kari sheep having litter sizes 1 and 2.

The genetic distance analysis showed that the ZP3 gene makeup of Kari having litter size 1 is the most distant as compared to other sheep breeds (Figure 5). Table 9 show genetic distances between the three sheep breeds. The result showed that the genetic distance based on ZP3 gene polymorphism in Kari having litter size 1 is more with Balkhi and Madakhlasht, compared to the distance with Kari having litter size 2. Madakhlasht sheep showed the least genetic distance with Balkhi.

**Table 9.** Genetic distance among the three sheep breeds.

	Kari		
	Litter size 1	Litter size 2	Madakhlasht
Kari (litter size 2)	0.3597		
Madakhlasht	0.4555	0.3261	
Balkhi	0.5421	0.2935	0.1599

### Population differentiation (F_ST_)

Genetic differentiation based on ZP3 gene polymorphism among sheep having litter size one and litter size two was evaluated using POPGENE software (Table 10). The results showed that Kari having litter size 2 and Kari having litter size 1 possess high (25%) differentiation at the exonic region and low (18%) at the upstream region. The overall differentiation between Kari having litter sizes 1 and 2 was 31.3%. Population differentiation between Kari having litter size 2 and Madakhlasht was higher (25.6%) at the upstream region and low (3.6%) at the exonic region, with an overall differentiation of 14.9%. Population differentiation between Kari with litter size 2 and Balkhi was also higher (29.3%) at the upstream region and low (11.0%) at the exonic region. Overall, the population differentiation between Kari having litter size 2 and Balkhi was 13.3%.

**Table 10.** Population differentiation (F_ST_) of Kari having litter size 2 with other breeds having litter size one.

Genic region	Kari2 vs Kari 1	Kari2 vs Madakhlasht	Kari2 vs Balkhi
Upstream	0.183	0.256	0.293
Intronic	0.203	0.149	0.111
Exonic	0.256	0.036	0.11
Overall	0.314	0.149	0.133

### Protein remodelling

**Figure 6.**
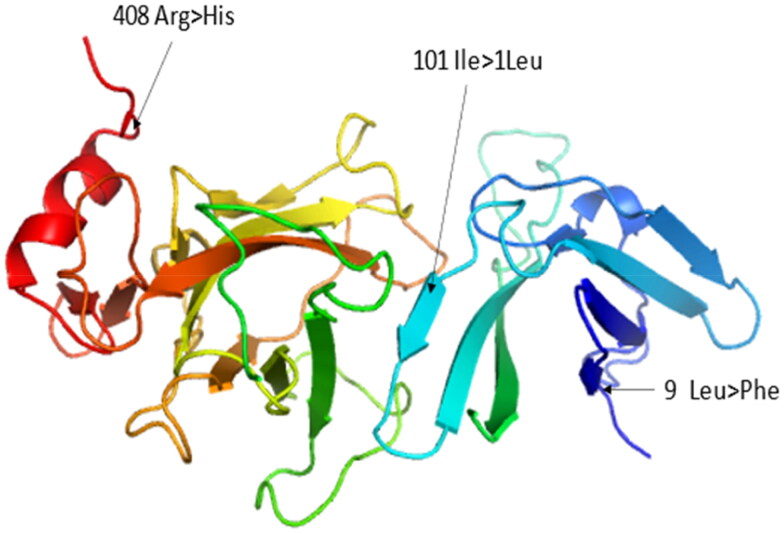
Protein modeling showing the position of the amino acid change in 3D structure. Association of SNPs with litter size and udder morphology

The positions of amino acid changes in the mutated protein model of the ZP3 protein is presented in Figure 6. The SNP at position 35135342 changing amino acid Leu9Phe and at position 35142968 changing amino acid Arg408His did not show any effect on the structure of ZP3 protein. However, the SNP at position 35135618 changing amino acid Ile101Leu showed cavity altered effect on protein structure (The substitution leads to an expansion or contraction of the cavity volume of ≥ 70 Å^3.) changing the binding site of the protein.

**Figure 7.**
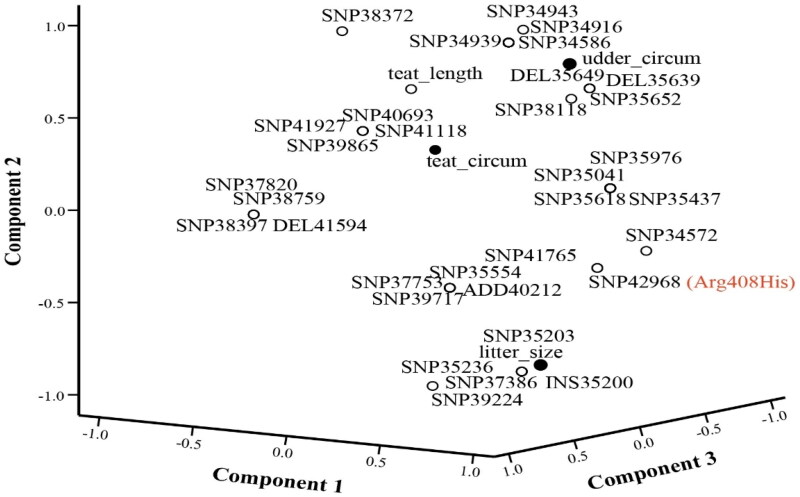
Principle component analysis plot showing SNPs associated with litter size and udder morphometry.

The result of principal component analysis (Figure 7) shows potential variants correlated with litter size in the three sheep breeds. As seen in the component plot SNPs at locus 35135203, 35135236, 35137386, and insertion at locus 35135200 in the upstream region of the gene-positive correlation with litter size in sheep. These variants are only found in Kari having litter size 2. SNP at locus 35142968 having an amino acid change (Arg408His) also correlated significantly with the litter size. The intronic SNP at locus 35139224 also sowed to affect the litter size. This mutation was observed in a heterozygous state in Kari having litter size 2, while homozygous in Kari having litter size 1. Intronic deletions at genomic positions 35135649 and 35135639 are positively correlated with udder circumference in Madakhlasht. These mutations are only present in Madakhalsht. Similarly, intronic deletion at genomic position 35135652 and SNP at genomic position 35134586 is related to udder circumference. These SNP were present in all three sheep breeds.

### Ewens Watterson test for neutrality

**Figure 8.**
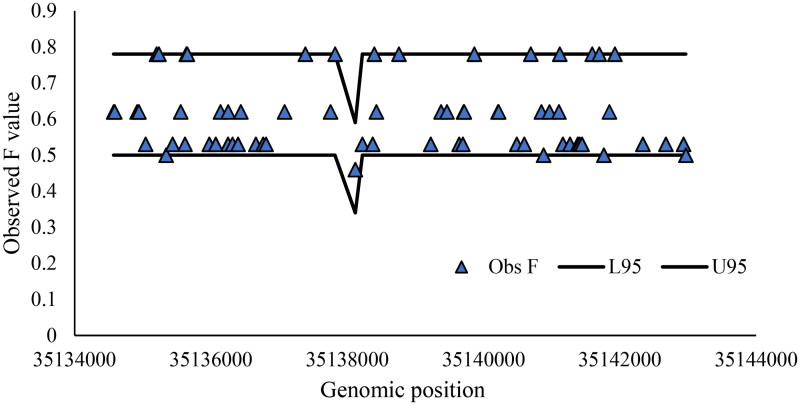
Ewens Watterson test for the neutrality of the mutated loci. The two lines show upper and lower 95% confidence intervals.

All the variants identified in the ZP3 gene of the sheep breeds were neutral for selection showed by Ewens Watterson test for neutrality (Figure 8). The f values observed were within the 95% confidence interval, indicating that the mutations were not influenced by selection pressure

## Discussion

In this study, Balkhi sheep, which exclusively produced singletons, exhibited larger body sizes, while smaller-sized Kari and Madakhlasht sheep breeds occasionally showed twin births. Previous research has indicated that Dorset sheep have a body weight of 80.1 kg, heavier than Romanov sheep (74.3 kg), yet Dorset’s litter size is reported to be 1.77 ± 0.04, less than that of Romanov sheep (3.57 ± 0.07).^30^^,^^31^ Our findings align with this pattern, as Balkhi sheep, with heavier bodyweights, displayed smaller litter sizes compared to Kari and Madakhlasht sheep with smaller body sizes. Similarly, a study demonstrated that small-sized Chinese Hu sheep (average bodyweight 38.20 ± 3.05 kg) have smaller litter sizes compared to Dorset sheep (litter size 1.72).^21^^,^^32^ These results suggest that body size may not necessarily affect litter size.

In a previous study by^33^ the mean body length observed for Balkhi sheep (75.4 ± 0.78 cm) was the highest compared to Hashtnagri (73.0 ± 0.65 cm) and Michni (68.1 ± 0.51 cm) breeds. Their litter sizes were reported as 1.1 ± 0.02, 1.1 ± 0.02, and 1.05 ± 0.01, respectively,^34^ indicating that Balkhi, despite its larger size, had a higher litter size compared to Michni. However, in the current study, Balkhi sheep with a longer body length (79 ± 1.8 cm), displayed a smaller litter size.

Udder morphology reveals a greater udder circumference in Madakhlasht sheep, which have occasionally exhibited twin births, while Balkhi sheep, which produce singletons, have a smaller udder circumference. A previous study has^35^ demonstrated that udder circumference is higher in Ghezel compared to Meharban sheep. Additionally, although Balkhi sheep have longer teats, they produce singletons, whereas Kari sheep with shorter teats have twins. Similarly, the teat length of Ghezel sheep is higher compared to Meharban sheep.^35^

In a previous study by^36^ it was highlighted that the ZP3 gene plays a crucial role in animal reproduction, with ZP3 being the only well-documented example of oocyte-specific gene expression in mammals. They demonstrated direct expression of a reporter gene (encoding firefly luciferase) in the upstream region of the mouse ZP3 gene, specifically in growing oocytes of transgenic mice harbouring the ZP3/luciferase construct. In the current study, nucleotide polymorphisms were observed in the upstream region at genomic positions 35135203, 35135236, and 35137386, along with an insertion of five nucleotides, showing variation among Kari sheep with different litter sizes. These variations in the upstream region may influence the expression of the ZP3 protein, ultimately impacting litter size in these sheep breeds.

In addition to the variation observed in the upstream region of the ZP3 gene, three missense single nucleotide polymorphisms (SNPs) in the coding region of the ZP3 gene were identified in the current study. These findings align with previous research, such as a study where a heterozygous mutation (p.Ser173Cys, c.518C > G) in the ZP3 gene was detected in a patient with empty follicle syndrome, impacting ZP structure.^37^ Disulfide analysis indicated that the S173C mutation may disrupt the stability of disulfide bonds and affect interactions between ZP3 and other ZP proteins. Additionally, five SNPs (rs422747079, rs407233145, rs420213968, rs401271989, rs398744310) have been previously reported, with the SNP (g.2293C > T) showing significant effects on litter size in heterozygous condition.^22^

In the current study, three single nucleotide polymorphisms (SNPs) were identified in the coding region, resulting in changes to the amino acid sequence of the ZP3 protein. Two of these SNPs, rs401271989 (c.301A > C) Ile1011Leu and rs398744310 (c.1223G > A) 408 Arg > His, were previously reported in a study by^22^ to be associated with amino acid changes. The authors suggested that the Ile to Leu mutation might affect the function of the ZP3 protein by influencing secondary and tertiary protein structures, correlating this specific mutation with litter size in Hu sheep. However, in contrast to that study, our current study did not find any correlation between the Ile1011Leu mutation and litter size, as this mutation was observed in all three sheep breeds. Instead, the mutation in Exon 8 (Arg408His), found only in sheep with a litter size of 2, may have a more significant effect on litter size based on our findings. Additionally, one novel SNP, c.25C > T, resulting in an amino acid change from Leu to Phe, was identified in all three sheep breeds in a heterozygous form.

The three-dimensional structural model of the ZP3 protein in sheep indicated that the amino acid substitution Ile1011Leu is located at the beginning of a β-strand, suggesting a potential ligand-binding site. Previous research has demonstrated that this β-strand is preceded by a single-turn α-helix and forms a β-bulge with a serine in the subsequent β-strand.^38^ Consistent with this finding, predictions of transmembrane regions revealed that the substitution of isoleucine with leucine occurs at the receptor site outside of the zona pellucida membrane, reinforcing the evidence that this locus may serve as a ligand-binding site.^22^

## Conclusions

Bodyweight has negative effect on litter size, sheep with larger body have a smaller litter size than sheep with lighter bodyweight. Teat length and circumference were greater in sheep with higher litter size. Six SNP in the upstream region of the ZP3 gene were positively correlated with litter size in Kari sheep. Among the three missense mutations the SNP (c.1223G > A) Arg408His can be used as a selection marker to improve the litter size in sheep. Further expression studies are required to confirm the effect of upstream variants on gene expression.

## Supplementary Material

supplementary tables.docx

## Data Availability

On request
