# Supplementary material for: Genetic variation in zona pellucida-3 (ZP3) gene and its association with litter size variation in Kari sheep
Source: Anim Biotechnol. 2025 Jan 24;36(1):2450364. doi: 10.1080/10495398.2025.2450364 (PMC12674277; doi:10.1080/10495398.2025.2450364)
Supplement: supplementary tables.docx [file LABT_A_2450364_SM1060.docx]

Supplementary table 1: Intron variants of the ZP3 gene among sheep breeds.

| **position** | **Reference** | **Kari-1** | **kari-2** | **Madakhlasht** | **Balkhi** | **Mutation frequency** |
| --- | --- | --- | --- | --- | --- | --- |
| 35135639 | ACCCCCTCG |  |  | A:ACCCCCTCG |  | 0.125 |
| 35135649 | CAGT |  |  | C:CAGT |  | 0.125 |
| 35135652 | T | C:C | C:C | C:T | C:C | 0.875 |
| 35135976 | C | T:T | T:C | T:C | T:C | 0.625 |
| 35136069 | GA | G:G | G:GA | G:GA | G:GA | 0.625 |
| 35136139 | C | A:A | A:A | A:C | A:C | 0.750 |
| 35136250 | A | C:C | C:A | C:A | C:A | 0.625 |
| 35136253 | G |  |  | GC:G | GC:G | 0.250 |
| 35136315 | A | G:G | G:A | G:A | G:A | 0.625 |
| 35136397 | C | T:T | T:C | T:C | T:C | 0.625 |
| 35136437 | G | C:C | C:C | C:G | C:G | 0.750 |
| 35136658 | T | C:C | C:T | C:T | C:T | 0.625 |
| 35136765 | G | A:A | A:G | A:G | A:G | 0.625 |
| 35136808 | A | G:G | G:A | G:A | G:A | 0.625 |
| 35136952 | A | G:G | G:G | G:G | G:G | 1.00 |
| 35137080 | G | C:C | C:C | C:G | C:G | 0.750 |
| 35137386 | G |  | A:G |  |  | 0.125 |
| 35137753 | C |  | T:C |  | T:C | 0.250 |
| 35137820 | C | CA:C |  |  |  | 0.125 |
| 35138118 | C | A:A | A:C | A,T:C | A,T:C | 0.875 |
| 35138222 | C | T:T | T:C | T:C | T:C | 0.625 |
| 35138372 | T | C:T |  | C:T | C:T | 0.375 |
| 35138397 | G | A:G |  |  |  | 0.125 |
| 35138427 | AGAG |  |  | A:AGAG | A:AGAG | 0.250 |
| 35139224 | G | A:A | A:G |  |  | 0.375 |
| 35139377 | A | G:G | G:G | G:A | G:A | 0.750 |
| 35139462 | G |  |  | C:G | C:G | 0.250 |
| 35139642 | C | T:T | T:C | T:C | T:C | 0.625 |
| 35139696 | T | G:G | G:T | G:T | G:T | 0.625 |
| 35139706 | T |  |  | C:T | C:T | 0.250 |
| 35139717 | T | C:C | C:T | C:C | C:T | 0.750 |
| 35139865 | C |  |  |  | T:C | 0.125 |
| 35140212 | G | GAT:GAT | GAT:G | GAT:GAT | GAT:G | 0.750 |
| 35140220 | T | C:C | C:T | C:C | C:T | 0.750 |
| 35140488 | T | A:A | A:T | A:T | A:T | 0.625 |
| 35140596 | A | G;G | G:A | G:A | G:A | 0.625 |
| 35140693 | C |  |  |  | T:C | 0.125 |
| 35140847 | A |  |  | T:A | T:A | 0.250 |
| 35140879 | A | AGCCTGACCTCTGGGAAGGCT:A | AGCCTGACCTCTGGGAAGGCT:A | AGCCTGACCTCTGGGAAGGCT:A | AGCCTGACCTCTGGGAAGGCT:A | 0.5 |
| 35140968 | A |  |  | G:A | G:A | 0.20 |
| 35141105 | A |  |  | AGTG:A | AGTG:A | 0.250 |
| 35141118 | G |  |  |  | A:G | 0.125 |
| 35141164 | C | T:T | T:C | T:C | T:C | 0.625 |
| 35141268 | A | G:G | G:A | G:A | G:A | 0.625 |
| 35141382 | T | C:C | C:T | C:T | C:T | 0.625 |
| 35141411 | C | A:A | A:C | A:C | A:C | 0.625 |
| 35141445 | A | T:T | T:A | T:A | T:A | 0.625 |
| 35141594 | CTTCT | C:CTTCT |  |  |  | 0.125 |
| 35141698 | C |  |  | T:C |  | 0.125 |
| 35141845 | T |  | C:T |  | C:T | 0.250 |
| 35142338 | A | G:G | G:A | G:A | G:A | 0.625 |
| 35142675 | C | T:T | T:C | T:C | T:C | 0.625 |

Grey boxes indicate homozygous variants.
